
JOURNAL INFORMATION
==============================
NLM Title Abbreviation: ScientificWorldJournal
Journal ID: ScientificWorldJournal
NLM Journal ID: 101131163
Journal ID: TSWJ

The Scientific World Journal

ISSN: 2356-6140
EISSN: 1537-744X
Publisher: Wiley

ARTICLE INFORMATION
==============================
PMCID: PMC5900850
PMID: 18040544
DOI: 10.1100/tsw.2007.285
Article ID: 463946
Article version: 1
Subjects: Directions in Science

Heparan Sulfate Proteoglycan–Involving Immunomodulation by Cathelicidin Antimicrobial Peptides LL-37 and PR-39

Kaneider Nicole C. 1
Djanani Angela 1
Wiedermann Christian J. 2 *
1 Division of General Internal Medicine, Department of Internal Medicine, Medical University of Innsbruck, Austria
2 2 nd Division of Internal Medicine, Department of Internal Medicine, Central Hospital of Bolzano, Italy
*Christian J. Wiedermann: christian.wiedermann@asbz.itAcademic Editor: Mauro Perretti

Publication date: 2007
Electronic publication date: 2007 Nov 12
Volume: 7
First page: 1832
Last page: 1838
Copyright: Copyright © 2007 Nicole C. Kaneider et al.
Copyright year: 2007
License: This is an open access article distributed under the Creative Commons Attribution License, which permits unrestricted use, distribution, and reproduction in any medium, provided the original work is properly cited.
License URL: https://creativecommons.org/licenses/by/3.0/

Keywords: inflammation, host defense, glycosaminoglycans, cell-matrix interaction, cell signalling

==============================
Directions in Science
*TheScientificWorldJOURNAL* (2007) 7, 1832–1838
ISSN 1537-744X; DOI 10.1100/tsw.2007.285

TheScientificWorldJOURNAL
www.thescientificworld.com

### Heparan Sulfate Proteoglycan–Involving Immunomodulation by Cathelicidin Antimicrobial Peptides LL-37 and PR-39

Nicole C. Kaneider[1], Angela Djanani[1], and Christian J. Wiedermann[2,]*

*[1]Division of General Internal Medicine, Department of Internal Medicine, Medical University of Innsbruck, Austria; [2]2nd Division of Internal Medicine, Department of Internal Medicine, Central Hospital of Bolzano, Italy*

**E-mail:** christian.wiedermann@asbz.it

*Received September 17, 2007; Revised October 14, 2007; Accepted October 17, 2007; Published November 12, 2007*

**KEYWORDS**: inflammation, host defense, glycosaminoglycans, cell-matrix interaction, cell signalling

Antimicrobial peptides, which are categorized by homologous structural motifs, are effector molecules of innate immunity with direct antimicrobial activity and multiple other functions. The most prominent families are defensins and cathelicidins. These peptides are expressed in the granules of several types of leukocytes and in a wide variety of tissue types[1]. Peptide antibiotics of the cathelicidin family are characterized by a highly conserved signal sequence and proregions ("cathelin" = cathepsin L inhibitor), but show substantial heterogeneity in the carboxy-terminal domain that encodes the mature peptide[2,3,4]. Based on amino acid sequences, cathelicidin peptides are categorized in (a) linear, a-helical peptides without cysteines, e.g., LL-37/human cathelicidin antimicrobial peptide (hCAP)-18 from human; (b) peptides with an even number of cysteines linked by disulfide bridges, e.g., protegrins (porcine cathelicidin peptides); and (c) peptides with an unusually high proportion of one or two amino acids, e.g., PR-39 from porcine leukocytes (Table 1).

**TABLE 1**
**Selected Members of the Cathelicidin Family of Antimicrobial Peptides***

| Name | Source | Sequence | No. of Amino Acids | Hydrophobic Percentage | Protein Binding Potential (kcal/mol) |
|---|---|---|---|---|---|
| LL-37 | Human | LLGDFFRKSKEKIGKEFKRIVQRIKDFLRNLVPRTES | 37 | 35% | 2.99 |
| PR-39 | Pig | RRRPRPPYLPRPRPPPFFPPRLPPRIPPGFPPRFPPRFP | 39 | 20% | 3.04 |

* Retrieved from http://aps.unmc.edu/AP/main.php, the Antimicrobial Peptide Database of the Eppley Institute, University of Nebraska Medical Center (accessed Oct 15, 2007).

*Corresponding author.
©2007 with author.
Published by TheScientificWorld; www.thescientificworld.com

The ability of cathelicidins to inhibit growth of bacteria is the most prominent function of these molecules. The heterogenous carboxy-terminal domain represents the antimicrobially active peptide[1]. Host ionic conditions dictate microbial sensitivity to antimicrobial peptides[5]. After electrostatic interactions between the negatively charged microbial surface and the positively charged peptides, the peptides associate with the membranes, leading to a destabilization of the membrane and subsequent microbial. Several cathelicidin peptides bind to bacterial endotoxin (LPS) and inactivate its biological activity[1]. Bacterial contact with epithelial cells results in rapid production and secretion of the respective peptides[6], and, e.g., in the urinary tract, epithelium-derived cathelicidin substantially helps in the protection against infection[7]. Neutrophils also contribute substantially to innate resistance to infection with *Mycobacterium tuberculosis* bacilli, an activity associated with their antimicrobial peptides[8], suggesting that elucidation of the regulation of neutrophil antimicrobial peptides could facilitate prevention and treatment of tuberculosis.

Cathelin-related antimicrobial peptides have emerged as effector substances of the innate immune system involving not only activities as endogenous antibiotics, but also as mediators of inflammation, wound healing, and tissue repair (Fig. 1). Direct microbe killing by some antimicrobial peptides is prevented by physiological conditions, including high monovalent or moderate divalent cation concentrations, host proteases, low local peptide concentrations, and polyvalent anions, such as heparan sulfate proteoglycans (HSPGs)[9,10]. Conversely, these peptides are important effector molecules of the innate immune system[1,11] by mechanisms that may involve HSPGs. The role of cathelicidins in modulating the innate immune response and boosting infection-resolving immunity while dampening potentially harmful proinflammatory (septic) responses gives these peptides the potential to become an entirely new therapeutic approach against bacterial infections. Antimicrobial peptides are able to enhance phagocytosis, stimulate prostaglandin release, neutralize the septic effects of LPS, promote recruitment and accumulation of various immune cells at inflammatory sites[12,13], promote angiogenesis[14], and induce wound repair[15]. Peptides of mammalian origin have also been demonstrated to have an active role in the transition to the adaptive immune response by being chemotactic for human monocytes[16] and T cells[17], and by exhibiting adjuvant and polarizing effects in influencing dendritic cell development[18].

The human cathelicidin peptide LL-37, consisting of the 37 C-terminal amino acids hCAP-18 (SwissProt ID: P49913), is chemotactic for neutrophils, monocytes, mast cells, and T cells; induces degranulation of mast cells; alters transcriptional responses in macrophages; and stimulates wound vascularization and re-epithelialization of healing skin. The porcine PR-39 has also been involved in a variety of processes, including promotion of wound repair, induction of angiogenesis, neutrophils chemotaxis, and inhibition of the phagocyte NADPH oxidase activity, whereas a bovine host defense peptide, BMAP-28, induces apoptosis in transformed cell lines and activated lymphocytes and may thus help with clearance of unwanted cells at inflammation sites[3].

The basic biophysical understanding of antimicrobial peptides is still very limited[19]. This is especially unsatisfactory since knowledge of structural properties will greatly help in the understanding of their immunomodulatory functions. The mechanisms involved in direct immunomodulatory actions of cathelicidins are poorly characterized. Mechanisms may well be as heterogenous as are modes of their antimicrobial actions. As examples, the peptide LL-37 binds to formyl peptide receptor-like 1 (FPRL1), a G protein–coupled, seven transmembrane, cell receptor found on various cell types, including macrophages, neutrophils, and subsets of lymphocytes that are functionally activated by endogenous anti-inflammatory mediators, lipoxin A4 and annexin 1[20]. The receptor is activated by high concentrations of N-formyl-methionyl-leucyl-phenylalanine (fMLP) and several endogenous and exogenous ligands, and likely mediates cellular effects of LL-37, such as chemoattraction of neutrophils, monocytes, and T cells[20], as well as the activation of mast cells[21]. LL-37 has been implicated as a mediator of inflammation through modulating chemokine and cytokine production by macrophages, histamine release from mast cells[22], and chemokine release from epithelial cells[23,24,25]. To date, several receptors associated with LL-37–mediated immunomodulation have been identified, including FPRL1[20], purinergic receptor P2X7[26], formyl peptide receptor[27], and epidermal growth factor receptor

(EGFR)[24,28]. Although growing evidence suggests that LL-37 acts in a receptor-dependent fashion, the exact mechanisms of how it exerts these nonantimicrobial functions are yet to be determined.

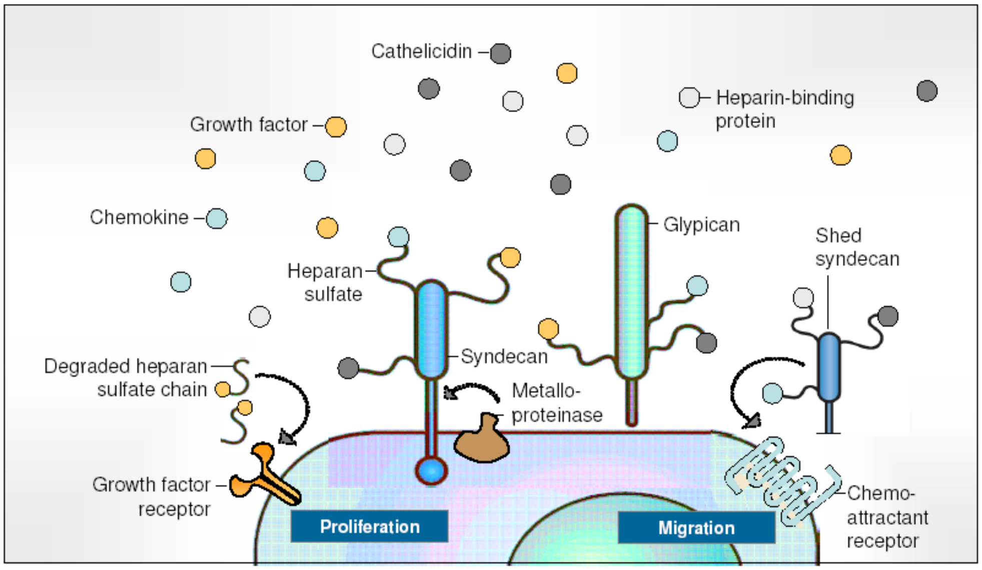

**FIGURE 1.** The role of HSPGs in mediating immunomodulatory effects of cathelicidins. Following an infectious stimulus, leukocytes and various types of tissue cells produce and release heparin-binding molecules, such as chemokines, growth factors, heparin-binding proteins, and antimicrobial peptides, such as cathelicidins, that bind with high affinity to host and bacterial binding sites, and associate with their diverse heparan sulfate–binding domains on matrix and cell surface proteoglycans. Ligand affinity and concentration determine competitive displacement of heparan sulfate–bound mediators. Activation or inhibition of proteases and heparanases alter constitutive shedding of HSPGs. Binding of cathelicidins to HSPG may alter focal adhesion kinase signaling of syndecan-4 directly; affect coreceptor function of syndecans and glypicans, e.g., by activation of metalloproteinase and syndecan ectodomain shedding or by modification of enzymatic degradation of heparan sulfates by heparanase; and transactivate surface receptors of inflammatory cells, including the formyl peptide receptor, formyl peptide receptor like-1, purinergic receptor, epidermal growth factor receptor, or chemokine receptors. Binding of cathelicidins to heparan sulfate and shedding of HSPGs from cell surfaces at the same time affects microbial virulence and antimicrobial effects of the peptides.

PR-39, the proline-rich antimicrobial peptide of 39 amino acids (SwissProt ID: P80054) is also involved in several cellular processes, such as chemoattraction, angiogenesis, and inflammation[29,30,31,32]. Whether PR-39 utilizes different mechanisms than LL-37 in activating these functions is unknown. Antimicrobial mechanisms of action of PR-39 differ from other cathelicidin peptides. It crosses membranes rapidly and exerts its activity inside microbial cells by blocking bacterial DNA and protein synthesis[2,33]. The mature peptide is also capable of rapidly crossing eukaryotic cell membranes to bind to SH3 domains of the cytosolic component of the NADPH complex protein p47phox and the signaling adapter protein p130cas[34]. PR-39 is chemoattractive for neutrophils in a calcium-dependent and pertussis toxin–inhibitable reaction and contributes to wound healing by stimulating the expression of syndecans, cell surface HSPGs[29]. Removal of HSPGs from the surface of neutrophils abrogates their migratory response to PR-39, but not to fMLP[30], suggesting that cell surface receptors are involved in PR-39 functions. Evidence also indicates that PR-39 stimulates angiogenesis by inhibiting the ubiquitin proteasome–dependent degradation of hypoxia-inducible factor 1-alpha protein; this selective inhibitory effect of the peptide in the proteasome leads to an anti-inflammatory activity by blocking the degradation of the NF-kB inhibitor IkBa[35]. PR-39 attenuates the apoptotic response of HeLa cells as well as other cell types[36,37,38]. These multiple biological activities of PR-39 have been used to prevent postischemic microvascular dysfunction, partly through stabilization of hypoxia-inducible factor 1-alpha[39]. PR-39 has also been identified in human skin–wound fluid and has been shown to increase levels of the cell surface HSPGs syndecan-1 and -4 in NIH 3T3 fibroblasts[40]. PR-39–mediated alterations of cell adhesion and motility are related, in part, to the increased expression of cell surface syndecan-4, a finding that supports the notion that factors that control syndecan-4 expression may play an important role in regulating adhesion-related cell processes[41]. Increased expression of syndecan-1 in hepatocellular carcinoma cells by PR-39 was associated with suppression of motile activity and alteration of actin structure in addition to the suppression of the carcinoma cells' invasive activity[42]. In PR-39–induced migration of neutrophils, modification of HSPGs with sodium chlorate inhibited migration, whereas chemotaxis toward the chemoattractant fMLP was not affected, and removal of heparan sulfates or chondroitin sulfates from the surface of neutrophils by heparinase or chondroitinase inhibited migration toward PR-39, indicating participation of HSPGs of neutrophils — likely syndecans, as both heparinase and chondroitinase were abrogating — in cathelicidin peptide–mediated regulation of the antimicrobial host defense[30]. These observations tightly link HSPGs and PR-39 in cathelicidin-induced immunomodulatory functions.

The polysaccharide heparan sulfate is ubiquitously expressed as a proteoglycan in extracellular matrices and on cell surfaces that function as receptors/coreceptors of extracellular ligands[43], including many microbes[44]. Among the multiple roles of heparan sulfate in inflammatory responses, it participates in almost every stage of leukocyte transmigration through the blood vessel wall. Heparan sulfate is involved in the initial adhesion of leukocytes to the inflamed endothelium, the subsequent chemokine-mediated transmigration through the vessel wall and the establishment of inflammatory reactions. Because of their great structural diversity, a wide range of proteins, such as chemokines, growth factors, and antimicrobial peptides, can bind to the hot spots of heparan sulfate chains, with many proteins binding to unique saccharide sequences. It presents CXC-chemokine ligand 8 (CXCL8) produced by tissue-resident macrophages, to chemokine receptors on leukocytes[43]. All chemokines seem to have the ability to interact with heparan sulfate on the surface of cells and in the extracellular matrix[45]. Human antimicrobial peptides, including LL-37, bind to HSPGs and, *vice versa*, structural motifs associated with heparin affinity (cationicity, amphipaticity, and consensus regions) confer antimicrobial properties to a given peptide[46]. When liberated from HSPGs by endoglucosidases, these heparin-binding polypeptide mediators are made available for tissue remodeling and angiogenesis, with heparan sulfate on the surface of target cells also being important for signaling through growth factor receptors[43].

The precise mechanisms, how cathelicidins may interfere with regulatory pathways, are unknown. Leukocyte and vascular HSPGs[43,47,48] may change the chemical structure of the heparan sulfate chains they express in response to cathelicidins and thereby alter the inflammatory response with changes in chemokine and growth factor binding known to be important for leukocyte function, angiogenesis, and tissue remodeling, but little is known about this possibility. Furthermore, regulation of heparin sulfate function can occur by changing the accessibility of the polysaccharide at sites of inflammation by protease-mediated shedding of HSPG ectodomains from cell surfaces[49,50,51]. Shedding of HSPGs can be regulated by growth factors and chemokines[52,53]. Interestingly, LL-37 induced keratinocyte migration via metalloproteinase-induced release of heparin-binding EGF from the cell surface that mediates transactivation of EGFR, which stimulates this migration[28]. Such data indicate that HSPG ectodomain shedding may also be regulated by cathelicidins, but further studies are needed. Last but not least, antimicrobial peptides could interact with heparan sulfate and mask ligand binding sites as do heparin sulfate–binding proteins, including antithrombin[54], platelet factor 4, and histidine-rich glycoprotein[55], but histidine-rich glycoprotein is also an inhibitor of heparanase activity[56].

Shed HSPGs, such as syndecan-1 ectodomains, may interfere with host defenses by interacting with different agents of the innate defense system. Purified syndecan-1 ectodomains, through their heparan sulfate chains, bind tightly to peptides of the cathelicidin family, such as PR-39, and inhibit their antibacterial activities[57]. Similar observations have been reported on shed dermatan sulfate–inactivating defensins[58]. Shed ectodomains also bind to neutrophil elastase and cathepsin G[59]; elastase, at least, has been shown to be important in defending the host against Gram-negative bacterial sepsis[60]. Finally, soluble heparan sulfate can inhibit the activity of several cytokines involved in phagocyte recruitment[61], implying that heparan sulfate chains of shed ectodomains may act similarly. Such inhibition of soluble, innate host defense factors may be one of the virulence factors of microbes[62], which activation of an effective innate host response to microbial injury normally would have to successfully counteract.

In summary, there is now ample evidence that the HSPGs participate in many aspects of inflammation. Following an infectious stimulus, leukocytes and various types of tissue cells produce and release heparin-binding molecules, such as chemokines, growth factors, heparin-binding proteins, and antimicrobial peptides, including cathelicidins that bind with high affinity to host or bacterial binding sites and associate with diverse heparan sulfate–binding domains of proteoglycans. Ligand affinity and concentration determine competitive displacement of heparan sulfate–bound mediators. Activation or inhibition of proteases and heparanases alter constitutive shedding of HSPGs. Binding of cathelicidins to HSPG may alter directly focal adhesion kinase signaling of syndecan-4; affect coreceptor function of syndecans and glypicans, e.g., by activation of metalloproteinase and shedding of syndecan ectodomains or by modification of enzymatic degradation of heparan sulfates by heparanase; and transactivate surface receptors of inflammatory cells, including formyl peptide receptor, FPRL1, purinergic receptor, EGFR, and chemokine receptors. At the same time, binding of cathelicidins to heparan sulfate and shedding of HSPGs from cell surfaces affects microbial virulence and the antimicrobial potential of the peptides Nevertheless, much still remains to be understood. For example, there are indications that cathelicidins induce liberation of heparin-binding EGF with subsequent transactivation of EGFR by activation of metalloproteinase[28], however, the role of cathelicidins in production and shedding of HSPGs by leukocytes, endothelial cells, and other types of cells is unknown. Presence of heparan sulfate–bound cathelicidins in the basement membrane and the effect of cathelicidins on production and release of heparanase used to solubilize HSPGs are unexplored. Furthermore, limited studies indicate that antimicrobial peptides specifically disrupt heparin sulfate–protein interactions. Regulation and molecular mechanisms of such interactions need to be studied in more detail to better understand how cathelicidins may be developed clinically as new anti-inflammatory agents. In conclusion, analysis of the participation of HSPGs in immunomodulatory effects of antimicrobial peptides has reached an interesting stage with various new directions of research now possible.

### REFERENCES

1. Bowdish, D.M., Davidson, D.J., and Hancock, R.E. (2006) Immunomodulatory properties of defensins and cathelicidins. *Curr. Top. Microbiol. Immunol.* **306**, 27–66.
2. Bals, R. and Wilson, J.M. (2003) Cathelicidins - a family of multifunctional antimicrobial peptides. *Cell Mol. Life Sci.* **60**, 711–720.
3. Zanetti, M. (2004) Cathelicidins, multifunctional peptides of the innate immunity. *J. Leukoc. Biol.* **75,** 39–48.
4. Zanetti, M. (2005) The role of cathelicidins in the innate host defenses of mammals. *Curr. Issues Mol. Biol.* **7**, 179–196.
5. Dorschner, R.A., Lopez-Garcia, B., Peschel, A., Kraus, D., Morikawa, K., Nizet, V., and Gallo, R.L. (2006) The mammalian ionic environment dictates microbial susceptibility to antimicrobial defense peptides. *FASEB J.* **20**, 35–42.
6. Wehkamp, J., Schauber, J., and Stange, E.F. (2007) Defensins and cathelicidins in gastrointestinal infections. *Curr. Opin. Gastroenterol.* **23**, 32–38.
7. Chromek, M., Slamova, Z., Bergman, P., Kovacs, L., Podracka, L., Ehren, I., Hokfelt, T., Gudmundsson, G.H., Gallo, R.L., Agerberth, B., and Brauner, A. (2006) The antimicrobial peptide cathelicidin protects the urinary tract against invasive bacterial infection. *Nat. Med.* **12**, 636–641.
8. Martineau, A.R., Newton, S.M., Wilkinson, K.A., Kampmann, B., Hall, B.M., Nawroly, N., Packe, G.E., Davidson, R.N., Griffiths, C.J., and Wilkinson, R.J. (2007) Neutrophil-mediated innate immune resistance to mycobacteria. *J. Clin. Invest.* **117**, 1988–1994.
9. Jenssen, H., Hamill, P., and Hancock, R.E. (2006) Peptide antimicrobial agents. *Clin. Microbiol. Rev.* **19**, 491–511.
10. Malmsten, M., Davoudi, M., Walse, B., Rydengård, V., Pasupuleti, M., Mörgelin, M., and Schmidtchen, A. (2007) Antimicrobial peptides derived from growth factors. *Growth Factors* **25**, 60–70.
11. Brown, K.L. and Hancock, R.E. (2006) Cationic host defense (antimicrobial) peptides. *Curr. Opin. Immunol.* **18**, 24–30.
12. Elsbach, P. (2003) What is the real role of antimicrobial polypeptides that can mediate several other inflammatory responses? *J. Clin. Invest.* **111**, 1643–1645.
13. Yang, D., Biragyn, A., Kwak, L.W., and Oppenheim, J.J. (2002) Mammalian defensins in immunity: more than just microbicidal. *Trends Immunol.* **23**, 291–296.
14. Koczulla, R., von Degenfeld, G., Kupatt, C., Krotz, F., Zahler, S., Gloe, T., Issbrucker, K., Unterberger, P., Zaiou, M., Lebherz, C., Karl, A., Raake, P., Pfosser, A., Boekstegers, P., Welsch, U., Hiemstra, P.S., Vogelmeier, C., Gallo, R.L., Clauss, M., and Bals, R. (2003) An angiogenic role for the human peptide antibiotic LL-37/hCAP-18. *J. Clin.*

*Invest.* **111**, 1665–1672.
15. Chan, Y.R. and Gallo, R.L. (1998) PR-39, a syndecan-inducing antimicrobial peptide, binds and affects p130(Cas). *J. Biol. Chem.* **273**, 28978–28985.
16. Territo, M.C., Ganz, T., Selsted, M.E., and Lehrer, R. (1989) Monocytechemotactic activity of defensins from human neutrophils. *J. Clin. Invest.* **84**, 2017–2020.
17. Chertov, O., Michiel, D.F., Xu, L., Wang, J.M., Tani, K., Murphy, W.J., Longo, D.L., Taub, D.D., and Oppenheim, J.J. (1996) Identification of defensin-1, defensin-2, and CAP37/azurocidin as T-cell chemoattractant proteins released from interleukin-8-stimulated neutrophils. *J. Biol. Chem.* **271**, 2935–2940.
18. Davidson, D.J., Currie, A.J., Reid, G.S., Bowdish, D.M., MacDonald, K.L., Ma, R.C., Hancock, R.E., and Speert, D.P. (2004) The cationic antimicrobial peptide LL-37 modulates dendritic cell differentiation and dendritic cell-induced T cell polarization. *J. Immunol.* **172**, 1146–1156.
19. Dürr, U.H.N., Sudheendra, U.S., and Ramamoorthy, A. (2006) LL-37, the only human member of the cathelicidin family of antimicrobial peptides. *Biochim. Biophys. Acta* **1758**, 1408–1425.
20. De, Y., Chen, Q., Schmidt, A.P., Anderson, G.M., Wang, J.M., Wooters, J., Oppenheim, J.J., and Chertov, O. (2000) LL-37, the neutrophil granule- and epithelial cell-derived cathelicidin, utilizes formyl peptide receptor-like 1 (FPRL1) as a receptor to chemoattract human peripheral blood neutrophils, monocytes, and T cells. *J. Exp. Med.* **192**, 1069–1074.
21. Niyonsaba, F., Iwabuchi, K., Someya A, Hirata, M., Matsuda, H., Ogawa, H., and Nagaoka, I. (2002) A cathelicidin family of human antibacterial peptide LL-37 induces mast cell chemotaxis. *Immunology* **106**, 20–26.
22. Niyonsaba, F., Someya, A., Hirata, M., Ogawa, H., and Nagaoka, I. (2001) Evaluation of the effects of peptide antibiotics human beta-defensins-1/-2 and LL-37 on histamine release and prostaglandin D(2) production from mast cells. *Eur. J. Immunol.* **31**, 1066–1075.
23. Scott, M.G., Davidson, D.J., Gold, M.R., Bowdish, D., and Hancock, R.E. (2002) The human antimicrobial peptide LL-37 is a multifunctional modulator of innate immune responses. *J. Immunol.* **169**, 3883–3891.
24. Tjabringa, G.S., Aarbiou, J., Ninaber, D.K., Drijfhout, J.W., Sörensen, O.E., Borregaard, N., Rabe, K.F., and Hiemstra, P.S. (2003) The antimicrobial peptide LL-37 activates innate immunity at the airway epithelial surface by transactivation of the epidermal growth factor receptor. *J. Immunol.* **171**, 6690–6696.
25. Braff, M.H., Hawkins, M.A., Nardo, A.D., Lopez-Garcia, B., Howell, M.D., Wong, C., Lin, K., Streib, J.E., Dorschner, R., Leung, D.Y., and Gallo, R.L. (2005) Structure-function relationships among human cathelicidin peptides: dissociation of antimicrobial properties from host immunostimulatory activities. *J. Immunol.* **174**, 4271–4278.
26. Elssner, A., Duncan, M., Gavrilin, M., and Wewers, M.D. (2004) A novel P2X7 receptor activator, the human cathelicidin-derived peptide LL37, induces IL-1 beta processing and release. *J. Immunol.* **172**, 4987–4994.
27. Tjabringa, G.S., Ninaber, D.K., Drijfhout, J.W., Rabe, K.F., and Hiemstra, P.S. (2006) Human cathelicidin LL-37 is a chemoattractant for eosinophils and neutrophils that acts via formyl-peptide receptors. *Int. Arch. Allergy Immunol.* **140**, 103–112.
28. Tokumaru, S., Sayama, K., Shirakata, Y., Komatsuzawa, H., Ouhara, K., Hanakawa, Y., Yahata, Y., Dai, X., Tohyama, T., Nagai, H., Yang, L., Higashiyama, S., Yoshimura, A., Sugai, M., and Hashimoto, K. (2005) Induction of keratinocyte migration via transactivation of the epidermal growth factor receptor by the antimicrobial peptide LL-37. *J. Immunol.* **175**, 4662–4668.
29. Huang, H.J., Ross, C.R., and Blecha, F. (1997) Chemoattractant properties of PR-39, a neutrophil antibacterial peptide. *J. Leukoc. Biol.* **61**, 624–629.
30. Djanani, A., Mosheimer, B., Kaneider, N.C., Ross, C.R., Ricevuti, G., Patsch, J.R., and Wiedermann, C.J. (2006) Heparan sulfate proteoglycan-dependent neutrophil chemotaxis toward PR-39 cathelicidin. *J. Inflamm.* **3**, 14.
31. Li, J., Post, M., Volk, R., Gao, Y., Li, M., Metais, C., Sato, K., Tsai, J., Aird, W., Rosenberg, R.D., Hampton, T.G., Sellke, F., Carmeliet, P., and Simons, M. (2000) PR39, a peptide regulator of angiogenesis. *Nat. Med.*, **6**, 49–55.
32. Delfino, D., Cusumano, V.V., Tomasello, F., and Cusumano, V. (2004) Beneficial effects of antibacterial peptide PR-39 in a neonatal murine model of endotoxic shock. *New Microbiol.* **27**, 369-374.
33. Chan, Y.R., Zanetti, M., Gennaro, R., and Gallo, R.L. (2001) Anti-microbial activity and cell binding are controlled by sequence determinants in the anti-microbial peptide PR-39. *J. Invest. Dermatol.* **116**, 230–235.
34. Shi, J., Ross, C.R., Leto, T.L., and Blecha, F. (1996) PR-39, a proline-rich antibacterial peptide that inhibits phagocyte NADPH oxidase activity by binding to Src homology 3 domains of p47 phox. *Proc. Natl. Acad. Sci. U. S. A.* **93**, 6014–6018.
35. Gao, Y., Lecker, S., Post, M.J., Hietaranta, A.J., Li, J., Volk, R., Li, M., Sato, K., Saluja, A.K., Steer, M.L., Goldberg, A.L., and Simons, M. (2000) Inhibition of ubiquitin-proteasome pathway-mediated I kappa B alpha degradation by a naturally occurring antibacterial peptide. *J. Clin. Invest.* **106**, 439–448.
36. Ross, C.R., Ricevuti, G., and Scovassi, A.I. (2007) The antimicrobial peptide PR-39 has a protective effect against HeLa cell apoptosis. *Chem. Biol. Drug. Des.* **70**, 154–157.
37. Wu, J., Parungo, C., Wu, G., Kang, P.M., Laham, R.J., Sellke, F.W., Simons, M., and Li, J. (2004) PR39 inhibits apoptosis in hypoxic endothelial cells: role of inhibitor apoptosis protein-2. *Circulation* **109**, 1660–1667.
38. Ramanathan, B., Wu, H., Ross, C.R., and Blecha, F. (2004) PR-39, a porcine antimicrobial peptide, inhibits apoptosis: involvement of caspase-3. *Dev. Comp. Immunol.* **28**, 163–169.
39. Muinck, E.D., Nagy, N., Tirziu, D., Murakami, M., Gurusamy, N., Goswami, S.K., Ghatpande, S., Engelman, R.M.,

Simons, M., Das, D.K. (2007) Protection against myocardial ischemia-reperfusion injury by the angiogenic Masterswitch protein PR 39 gene therapy: the roles of HIF1alpha stabilization and FGFR1 signaling. *Antioxid. Redox. Signal*. **9**, 437–445.
40. Gallo, R.L., Ono, M., Povsic, T., Page, C., Eriksson, E., Klagsbrun, M., and Bernfield. M. (1994) Syndecans, cell surface heparan sulfate proteoglycans, are induced by a proline-rich antimicrobial peptide from wounds. *Proc. Natl. Acad. Sci. U. S. A.* **91**, 11035–11039.
41. Chon, J.H., Houston, M.M., Xu, C., and Chaikof, E.L. (2001) PR-39 coordinates changes in vascular smooth muscle cell adhesive strength and locomotion by modulating cell surface heparan sulfate-matrix interactions. *J. Cell. Physiol.* **189**, 133–143.
42. Ohtake, T., Fujimoto, Y., Ikuta, K., Saito, H., Ohhira, M., Ono, M., and Kohgo, Y. (1999) Proline-rich antimicrobial peptide, PR-39 gene transduction altered invasive activity and actin structure in human hepatocellular carcinoma cells. *Br. J. Cancer* **81,** 393–403.
43. Parish, C.R. (2006) The role of heparan sulphate in inflammation. *Nat. Rev. Immunol.* **6**, 633–643.
44. Rostand, K.S. and Esko, J.D. (1997) Microbial adherence to and invasion through proteoglycans. *Infect. Immun.* **65**, 1–8.
45. Handel, T.M., Johnson, Z., Crown, S.E., Lau, E.K., and Proudfoot, A.E. (2005) Regulation of protein function by glycosaminoglycans—as exemplified by chemokines. *Annu. Rev. Biochem.* **74**, 385–410.
46. Kaneider, N.C., Egger, P., Dunzendorfer, S., and Wiedermann, C.J. (2001) Syndecan-4 as antithrombin receptor of human neutrophils. *Biochem. Biophys. Res. Commun.* **287,** 42–46.
47. Kaneider, N.C., Reinisch, C.M., Dunzendorfer, S., Romisch, J., and Wiedermann, C.J. (2002) Syndecan-4 mediates antithrombin-induced chemotaxis of human peripheral blood lymphocytes and monocytes. *J. Cell Sci.* **115**, 227–236.
48. Ihrcke, N.S. and Platt, J.L. (1996) Shedding of heparan sulfate proteoglycan by stimulated endothelial cells: evidence for proteolysis of cell-surface molecules. *J. Cell Physiol.* **168**, 625–637.
49. Fitzgerald, M.L., Wang, Z., Park, P.W., et al. (2000) Shedding of syndecan-1 and -4 ectodomains is regulated by multiple signaling pathways and mediated by a TIMP-3-sensitive metalloproteinase. *J. Cell Biol.* **148**, 811–824.
50. Asundi, V.K., Erdman, R., Stahl, R.C., and Carey, D.J. (2003) Matrix metalloproteinase-dependent shedding of syndecan-3, a transmembrane heparan sulfate proteoglycan, in Schwann cells. *J. Neurosci. Res.* **73**, 593–602.
51. Wang, Z., Gotte, M., Bernfield, M., and Reizes, O. (2005) Constitutive and accelerated shedding of murine syndecan-1 is mediated by cleavage of its core protein at a specific juxtamembrane site. *Biochemistry* **44**, 12355–12361.
52. Ding, K., Lopez-Burks, M., Sanchez-Duran, J.A., Korc, M., and Lander, A.D. (2005) Growth factor-induced shedding of syndecan-1 confers glypican-1 dependence on mitogenic responses of cancer cells. *J. Cell Biol.* **171,** 729–738.
53. Charnaux, N., Sutton, A., Brule, S., and Gattegno, L. (2006) Regulated shedding of syndecan ectodomains by chemokines. *TheScientificWorldJOURNAL* **6**, 1037–1040.
54. Kaneider, N.C., Forster, E., Mosheimer, B., Sturn, D.H., and Wiedermann, C.J. (2003) Syndecan-4-dependent signaling in the inhibition of endotoxin-induced endothelial adherence of neutrophils by antithrombin. *Thromb Haemost.* **90**, 1150–1157.
55. Brown, K.J. and Parish, C.R. (1994) Histidine-rich glycoprotein and platelet factor 4 mask heparan sulfate proteoglycans recognized by acidic and basic fibroblast growth factor. *Biochemistry* **33**, 13918–13927.
56. Freeman, C. and Parish, C.R. (1997) A rapid quantitative assay for the detection of mammalian heparanase activity. *Biochem. J.* **325**, 229–237.
57. Andersson, E., Rydengard, V., Sonesson, A., Morgelin, M., Bjorck, L., and Schmidtchen, A. (2004) Antimicrobial activities of heparin-binding peptides. *Eur. J. Biochem*. **271,** 1219–1226.
58. Schmidtchen, A., Frick, I., and Bjorck, L. (2001) Dermatan sulphate is released by proteinases of common pathogenic bacteria and inactivates antibacterial alpha-defensin. *Mol. Microbiol.* **39**, 708–713.
59. Kainulainen, V., Wang, H., Schick, C., and Bernfield, M. (1998) Syndecans, heparan sulfate proteoglycans, maintain the proteolytic balance of acute wound fluids. *J. Biol. Chem.* **273**, 11563–11569.
60. Belaaouaj, A. et al. (1998) Mice lacking neutrophil elastase reveal impaired host defense against Gram negative bacterial sepsis. *Nat. Med.* **4**, 615–618.
61. Kuschert, G.S.V.V. et al. (1999) Glycosaminoglycans interact selectively with chemokines and modulate receptor binding and cellular responses. *Biochemistry* **38,** 12959–12968.
62. Park, P.W., Pier, G.P., Hinkes, M.T., and Bernfield, M. (2001) Exploitation of syndecan-1 shedding by *Pseudomonas aeruginosa* enhances virulence. *Nature* **411**, 98–102.

**This article should be cited as follows:**

Kaneider, N.C., Djanani, A., and Wiedermann, C.J. (2007) Heparan sulfate proteoglycan–involving immunomodulation by cathelicidin antimicrobial peptides LL-37 and PR-39. *TheScientificWorldJOURNAL* **7**, 1832–1838. DOI 10.1100/tsw.2007.285.
